# Editorial: Interactive robots for healthcare and participation

**DOI:** 10.3389/frobt.2025.1750188

**Published:** 2025-12-10

**Authors:** Sebastian Schneider, David Silvera-Tawil, Anna-Lisa Vollmer, Ann Majewicz Fey

**Affiliations:** 1 Human Media Interaction, University of Twente, Enschede, Netherlands; 2 Australian E-Health Research Centre, Commonwealth Scientific and Industrial Research Organisation (CSIRO), Sydney, NSW, Australia; 3 Interactive Robotics in Medicine and Care, Medical School OWL, Bielefeld University, Bielefeld, Germany; 4 Human-Enabled Technology Laboratory (HeRo), Texas Robotics, The University of Texas at Austin, Austin, TX, United States

**Keywords:** assistive robotics, rehabilitation, human-robot interaction, healthcare, co-design, socially-assistive robotics

## 1 Introduction

The convergence of robotics, artificial intelligence, and human-centered design is transforming healthcare and participation across diverse populations (Ghafurian et al., 2024; Maleki Varnosfaderani and Forouzanfar, 2024; Zhu et al., 2025). Interactive robots, ranging from surgical robots to empathetic healthcare assistants, are increasingly being deployed to address critical challenges in healthcare delivery, aging, mental health, and rehabilitation (Jung et al., 2025; Vandemeulebroucke et al., 2021; Hermann et al., 2024; Shen et al., 2025; Silvera-Tawil, , 2024). This Research Topic explores the multifaceted role of interactive robotics in enhancing care, promoting autonomy, and supporting participation, particularly in contexts of vulnerability and cognitive or physical decline.

### 1.1 Framing the challenge

Healthcare systems around the world are facing mounting pressure due to demographic shifts, workforce shortages, and the rising demand for personalized, high-quality care (Bernhard et al., 2024). As populations age and chronic conditions become more prevalent, the urgency for scalable, adaptive, and emotionally intelligent technologies grows. Interactive robots offer a promising solution: they can automate routine tasks, provide companionship, support rehabilitation, and facilitate mental health screening while maintaining a human-centered approach (Hermann et al., 2024; Shen et al., 2025; Silvera-Tawil, , 2024).

However, translating these innovations into everyday practice remains challenging. Ethical concerns, usability barriers, and cultural differences often hinder adoption (Olatunji et al., 2025; Rogers et al., 2022), while the slow transfer of research prototypes into real-world applications further compounds the problem. Effective integration requires not only technical excellence but also alignment with clinical workflows, user expectations, and organizational realities (Klemme et al., 2021).

Recent advances show that interactive robotics can significantly enhance patient engagement, improve job satisfaction for healthcare professionals, and boost operational efficiency (Chang et al., 2021; G et al., 2025). Realizing this potential requires close collaboration between researchers and practitioners to bridge the gap between innovation and everyday clinical practice (Gasteiger et al., 2022; Björling and Rose, 2019; Olatunji et al., 2024). This Research Topic responds to that need by presenting interdisciplinary contributions that demonstrate how interactive robots can be effectively designed, evaluated, and integrated into healthcare workflows. Despite these promising developments, a critical challenge remains: aligning technological capabilities with real-world requirements. The following section explores how contributions in this Research Topic address this gap through participatory design, clinician-informed development, and longitudinal deployment.

## 2 Contributions to the topic

### 2.1 Enhancing mental health screening and support

Ahmadi Majd et al. introduced a machine learning–based web application for screening Social Anxiety Disorder (SAD) using multimedia scenarios and emotion regulation strategies. Their tool achieved high accuracy and reliability, distinguishing between SAD and non-SAD individuals based on their use of suppression, avoidance, and reappraisal. The authors propose integrating this screening approach into socially-assistive robots to improve accessibility and engagement, particularly for individuals reluctant to seek traditional care.

### 2.2 Supporting mobility and safety in aging populations

Naderer et al. presents a smart robotic walking aid designed to reduce fall risk in older adults through real-time balance monitoring and corrective actuation. The system combines a wearable inertial measurement unit, PID-controlled actuators, and an electromechanical braking mechanism to detect and respond to postural imbalances. Experimental validation demonstrated rapid stabilization and user safety, highlighting the potential of robotics to enhance mobility and independence in aging populations.

### 2.3 Comfort, companionship, and structure in long-term care

Hofstede et al. explored the use of huggable integrated socially assistive robots (HI-SARs) in long-term care settings (eldercare, disability care, and rehabilitation). These robots combine emotional comfort, verbal interaction, and activity monitoring. Across three studies, HI-SARs were well-received, particularly to support clients with cognitive impairments such as dementia. The authors emphasize the importance of personalization, hygiene management, and proactive use of sensor data to enhance effectiveness.

### 2.4 Bridging the gap between innovation and implementation

Gebellí and Ros present a comprehensive *in situ* participatory design methodology for assistive robots in healthcare settings. Their 10-month study at an intermediate healthcare center culminated in a 2-month deployment of an autonomous patrolling and room monitoring robot to enhance patient safety. The iterative co-design process, involving healthcare staff and low-fidelity prototypes, revealed five distinct user personas and highlighted the importance of context-based requirements gathering, adaptive interaction design, and sustained engagement.

Oliva et al. extend this theme to speech-language pathology, employing a four-week asynchronous remote community (ARC) approach with licensed clinicians. Their findings highlight the need for expressive, multimodal communication, customizable behaviors, and strong ethical safeguards. The study reinforces the value of clinician-centered design and positions socially assistive robots (SARs) as tools to augment (not replace) therapeutic relationships.

Tang and Dou provide an economic perspective through a systematic review and meta-analysis of robotic surgery in older adults. While their results confirm the cost-effectiveness of robotic interventions when long-term outcomes and quality-adjusted life years (QALYs) are considered, they also identify high initial investment as a significant barrier to widespread adoption.

Finally, Nguyen and Saito examine the implementation of nursing care robots in Japan, emphasizing the critical role of hands-on training, user experience, and collaboration between developers and educators. Their study reveals that although care robots can improve operational efficiency and reduce staff stress, concerns about usability, cost, and emotional burden persist.

## 3 Broader implications and future directions

Together, these contributions underscore the transformative potential of interactive robots in healthcare and reveal critical priorities for future development. First, **personalization and adaptability** emerge as central themes: robots must be tailored or adaptable to individual needs, preferences, and cultural contexts. Features such as customizable voices, adaptive feedback, and scenario-based interaction enhance engagement and therapeutic effectiveness.

Equally important is ethical and practical integration. Concerns around data privacy, emotional attachment, and task replacement demand responsible innovation frameworks and co-design approaches that actively involve caregivers, clinicians, and patients. It is generally agreed that robots should complement (not replace) human relationships, ensuring emotional safety and trust.

Advances in multimodal interaction––combining speech, touch, motion, and sensor data––enable richer, context-aware experiences. At the same time, scalability and accessibility remain essential for extending care to underserved populations. Web-based tools and cost-effective robotic platforms can bridge gaps in rural communities and support individuals with social anxiety or mobility limitations.

Finally, longitudinal evaluation is critical. Several studies in this Research Topic highlight the need for extended deployments and iterative refinement to understand user behavior, system performance, and real-world impact. Sustained engagement and adaptive design processes will ensure that interactive robots evolve from promising prototypes into practical, widely adopted solutions.

## 4 Conclusion

Interactive robots are moving beyond research labs into homes, clinics, and care facilities, offering new ways to support health, participation, and wellbeing. This Research Topic demonstrates how thoughtful design, rigorous evaluation, and interdisciplinary collaboration can unlock their full potential. The challenge ahead is not only technological but deeply human: ensuring these systems act as partners in care, enhancing dignity, autonomy, and quality of life across diverse populations.

Achieving widespread adoption will require overcoming barriers related to cost, infrastructure, regulatory frameworks, and workforce readiness. Success depends on robust evidence of clinical impact, proactive change management, and strategies that align innovation with human-centered care. By addressing these factors, interactive robots can evolve from promising prototypes into trusted tools that transform healthcare delivery.
